# 594. A Phase I, Multicenter, Randomized, Double-blind, Placebo-controlled Single Dose, Dose-ranging Study to Evaluate the Safety, Tolerability, and Immunogenicity of Orally Administered Bivalent GI.1/GII.4 Norovirus Vaccine in Healthy Lactating Females ≥ 18 years Old and Their Breast-feeding Infants

**DOI:** 10.1093/ofid/ofae631.189

**Published:** 2025-01-29

**Authors:** Lam Nguyen, Susan Greco, Becca A Flitter, Molly Braun, Nicholas D’Amato, Sean N Tucker, James F Cummings, Colin A Lester, Darreann Carmela Hailey

**Affiliations:** Vaxart Inc, Rancho Palos Verdes, CA; Vaxart, Inc, South San Francisco, California; Vaxart Inc, Rancho Palos Verdes, CA; Vaxart, Inc, South San Francisco, California; Vaxart, South San Francisco, California; Vaxart, South San Francisco, California; VAXART, South San Francisco, California; Vaxart, Inc., South San Francisco, California; Vaxart, Inc., South San Francisco, California

## Abstract

**Background:**

There are no licensed vaccines to prevent norovirus (NoV) illness, a leading cause of gastroenteritis that can result in severe/fatal outcomes in infants. An effective NoV vaccine may target two leading genotypes causing human NoV infection worldwide, GI.1 and GII.4. Clinical trials have shown our bivalent nonreplicating adenoviral-vector based NoV vaccine candidate to be safe, well-tolerated, and highly immunogenic. Labayo et al. demonstrated NoV positive mothers with high breastmilk NoV antibodies had breastfed infants with less severe NoV disease; here, we will report the safety and immunogenicity of our bivalent GI.1/GII.4 NoV vaccine candidate in healthy, lactating females and their breast-feeding infants.

**Figure:**
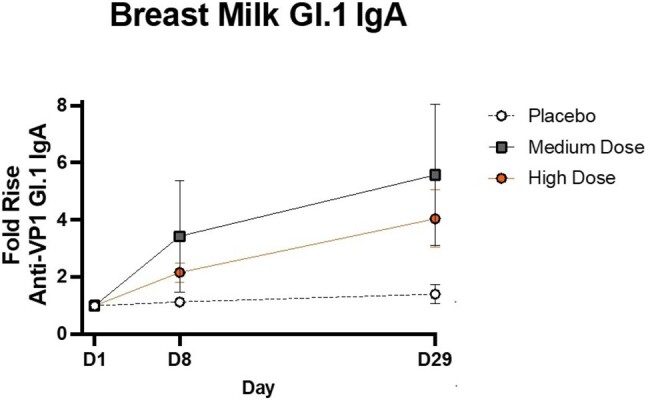

Graph 1. Breastmilk anti-VPI GI.1 IgA was quantified by MSD at Days 1, 8, and 29. Mean Fold Rises were evaluated from Day 1 to Day 1, from Day 1 to Day 8, and from Day 1 to Day 29. There is a progression of GI.1 IgA production during the first 28 days post-vaccination in the medium and high dose groups.

**Methods:**

76 lactating females ≥ 18 years old and their breast-feeding infants > 30 days to 11 months of age were randomized to receive a single oral dose of bivalent VXA-G1.1-NN and VXA-G2.4-NS at medium dose 1×10^11^ infectious units (IU) (n=30), high dose 2×10^11^ IU (n=30) or placebo (n=16). Primary safety endpoints include solicited adverse events (AEs) for one week post dose and unsolicited AEs for 28 days post dose.

Serum immunogenicity and breast milk antibodies were quantified by anti-VPI GI.1 and anti-VPI GII.4 IgA by Meso Scale Discovery (MSD) at Days 1, 8, and 29. Serum IgG was evaluated at the same timepoints.

**Figure:**
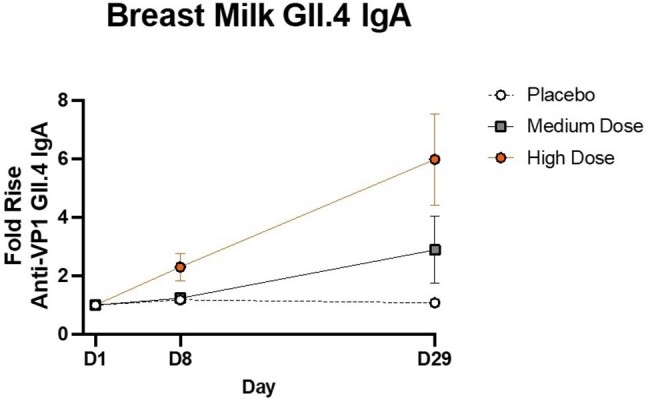

Graph 2. Breastmilk anti-VPI GII.4 IgA was quantified by MSD at Days 1, 8, and 29. Mean Fold Rises were evaluated from Day 1 to Day 1, from Day 1 to Day 8, and from Day 1 to Day 29. There is a progression of GII.4 IgA production during the first 28 days post-vaccination in the medium and high dose groups.

**Results:**

All doses of vaccine were well-tolerated. Solicited symptoms were similar to prior studies and without grade 3 or 4 events. There were no serious AEs reported to date.

Breast milk GI.1 IgA rose 4x above baseline in the high dose group on Day 29.

Breast milk GII.4 IgA rose 6x above baseline in the high dose group on Day 29.

Serum GI.1 IgA rose 3.4x for the medium dose and 2.7x for the high dose and GII.4 IgA rose 2x for the medium dose and 3.5x for the high dose on Day 29. Serum GI.1 IgG rose 3.4x for the medium dose and 5.1x for the high dose and GII.4 IgG rose 3.3x for the high dose on Day 29.

**Figure:**
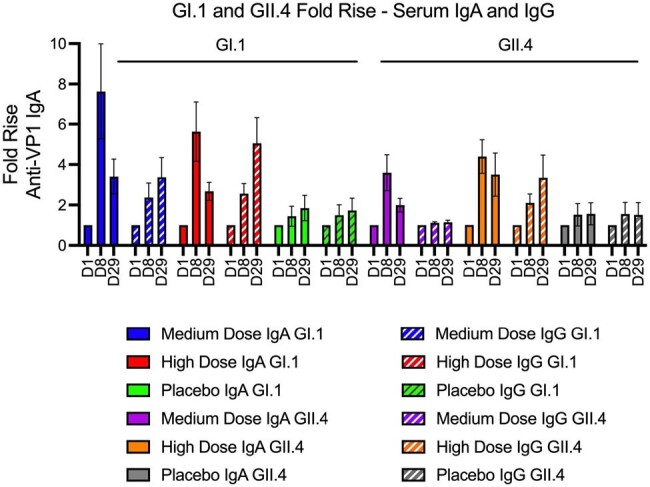

Graph 3. Serum immunogenicity antibodies were quantified by anti-VPI GI.1 IgA and IgG and anti-VPI GII.4 IgA and IgG by MSD at Days 1, 8, and 29. Mean Fold Rises were evaluated from Day 1 to Day 1, from Day 1 to Day 8, and from Day 1 to Day 29.

**Conclusion:**

Vaxart NoV bivalent vaccine candidate was safe and well-tolerated and elicited robust immune responses including the production of breastmilk NoV IgA in healthy, lactating female subjects. These results are an important step in the development of a NoV vaccine that is safe and immunogenic in lactating females with the potential to decrease disease severity in their breastfeeding infants.

VXA-NVV-108 Topline Immunogenicity Analysis

**Table 1.**
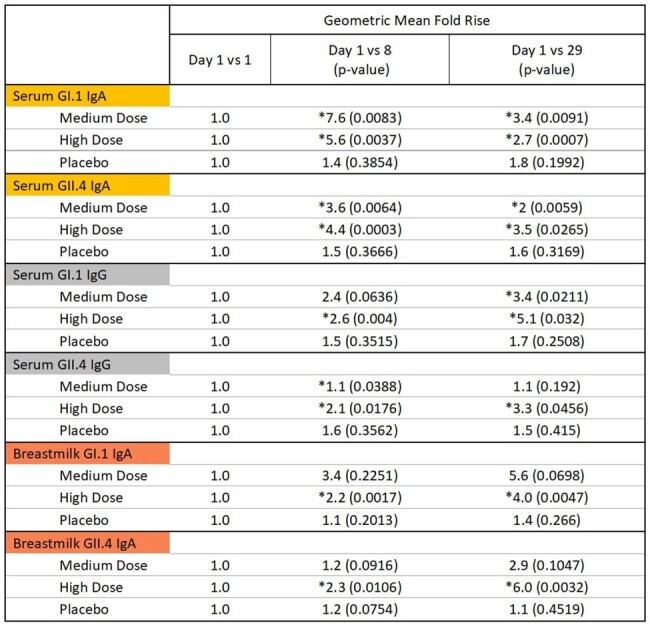
*Statistically significant Mean Fold Rise.

Serum and Breastmilk anti-VPI GI.1 IgA and anti-VPI GII.4 IgA are primary endpoints for the study. Serum anti-VPI GI.1 IgG and anti-VPI GII.4 IgG are a part of the secondary endpoints.

**Disclosures:**

**Lam Nguyen, MD**, CVS Health: Stocks/Bonds (Public Company)|Vaxart: Author is either employed and/or has received stock options from Vaxart as part of this work.|Vaxart: Stocks/Bonds (Public Company) **Susan Greco, MD, MPH**, Vaxart, Inc: Stocks/Bonds (Public Company) **Becca A. Flitter, PhD, MPH**, Vaxart Inc: Stocks/Bonds (Public Company) **Molly Braun, PhD**, Vaxart, Inc: TREATMENT OF LONG COVID WITH ORALLY AND MUCOSALLY ADMINISTERED ADENOVIRAL VECTORS|Vaxart, Inc: Stocks/Bonds (Public Company) **Nicholas D’Amato, MSc.**, Vaxart Inc.: Stocks/Bonds (Public Company) **Sean N. Tucker, PhD**, Vaxart, Inc.: Grant/Research Support|Vaxart, Inc.: patent|Vaxart, Inc.: Ownership Interest|Vaxart, Inc.: Stocks/Bonds (Public Company) **James F. Cummings, MD**, VAXART: Stocks/Bonds (Public Company) **Colin A. Lester, n/a**, Vaxart, Inc.: Stocks/Bonds (Public Company) **Darreann Carmela Hailey, MS**, Bionano Genomics: Stocks/Bonds (Public Company)|Vaxart, Inc.: Stocks/Bonds (Public Company)

